# Chromenone Derivatives as Monoamine Oxidase Inhibitors from Marine-Derived MAR4 Clade *Streptomyces* sp. CNQ-031

**DOI:** 10.4014/jmb.2105.05003

**Published:** 2021-05-31

**Authors:** Jong Min Oh, Chaeyoung Lee, Sang-Jip Nam, Hoon Kim

**Affiliations:** 1 Department of Pharmacy, and Research Institute of Life Pharmaceutical Sciences, Sunchon National University, Suncheon 57922, Republic of Korea; 2 Graduate School of Industrial Pharmaceutical Sciences, Ewha Womans University, Seoul 03760, Republic of Korea; 3 Department of Chemistry and Nanoscience, Ewha Womans University, Seoul 03760, Republic of Korea

**Keywords:** Monoamine oxidases, chromenone derivatives, *Streptomyces* sp. CNQ-031, reversible competitive inhibitors

## Abstract

Three compounds were isolated from marine-derived *Streptomyces* sp. CNQ-031, and their inhibitory activities against monoamine oxidases (MAOs), acetylcholinesterase (AChE), butyrylcholinesterase (BChE), and β-secretase (BACE-1) were evaluated. Compound 1 (5,7-dihydroxy-2-isopropyl-4H-chromen-4-one) was a potent and selective inhibitor of MAO-A, with a 50% inhibitory concentration (IC_50_) of 2.70 μM and a selectivity index (SI) of 10.0 versus MAO-B. Compound 2 [5,7-dihydroxy-2-(1-methylpropyl)-4H-chromen-4-one] was a potent and low-selective inhibitor of MAO-B, with an IC_50_ of 3.42 μM and an SI value of 2.02 versus MAO-A. Compound 3 (1-methoxyphenazine) did not inhibit MAO-A or MAO-B. All three compounds showed little inhibitory activity against AChE, BChE, and BACE-1. The K_i_ value of compound 1 for MAO-A was 0.94 ± 0.28 μM, and the K_i_ values of compound 2 for MAO-A and MAO-B were 3.57 ± 0.60 and 1.89 ± 0.014 μM, respectively, with competitive inhibition. The 1-methylpropyl group in compound 2 increased the MAO-B inhibitory activity compared with the isopropyl group in compound 1. Inhibition of MAO-A and MAO-B by compounds 1 and 2 was recovered by dialysis experiments. These results suggest that compounds 1 and 2 are reversible, competitive inhibitors of MAOs and can be considered potential therapies for neurological disorders such as depression and Alzheimer’s disease.

## Introduction

Monoamine oxidase (MAO; E.C. 1.4.3.4) is bound in the mitochondrial outer membrane and catalyzes the oxidative deamination of various primary, secondary, and tertiary amines, including monoamine neurotransmitter amines and therapeutic drugs [1]. MAO plays important roles in the pathways of catecholamine and 5-hydroxytryptamine inactivation. Inhibition of MAO offers antidepressant activities and behavioral benefits in the brain, so MAO is a major pharmaceutical target for drug design [1, 2]. MAO exists in two isoforms: MAO-A and MAO-B. Selective inhibitors of MAO-A are associated with antidepressive activity, whereas selective inhibitors of MAO-B have been used to treat Alzheimer’s disease (A_D_) and Parkinson’s disease (PD) [3]. MAO-A inhibitors preferentially remove serotonin, dopamine, and norepinephrine [4]. MAO-B is related to the inhibition of β-amyloid plaque formation associated with A_D_ [5]. Thus, various selective MAO-B inhibitors are being actively studied [6, 7] and have been reviewed recently [8, 9]. Furthermore, serious side effects of MAO inhibitors have not yet been reported [3].

Conversely, acetylcholinesterase (AChE; E.C. 3.1.1.7) has therapeutic efficacy in A_D_ by improving cholinergic transmission, which increases synaptic acetylcholine (ACh) levels in the cerebral cortex of patients with A_D_ [10]. Several studies have demonstrated the clinical benefits of sustained cholinesterase (ChE) inhibition by rivastigmine in A_D_ and PD. Rivastigmine has inhibitory activity against both AChE and butyrylcholinesterase (BChE; E.C. 3.1.1.8), whereas donepezil and galantamine selectively inhibit AChE [11]. Recently, therapeutic strategies have been devised using multitarget inhibitors that block both MAO and ChE; these studies demonstrated that MAO and AChE inhibitors can improve cognitive function and relieve A_D_ symptoms by increasing the levels of monoamines and choline esters [12-14]. In addition, β-secretase (BACE-1) inhibitors have been studied to treat A_D_ and PD because of their ability to inhibit β-amyloid accumulation [15].

Natural MAO inhibitors have been isolated and investigated from microbial sources, including aplysinopsins from *Aplysinopsis* sp. [16], piloquinones from *Streptomyces* sp. [17], anithiactins from *Streptomyces* sp. [18], and 5-hydroxy-2-methyl-chroman-4-one (HMC) from the endogenous lichen fungus *Daldinia fissa* [19]. Marine natural MAO inhibitors in particular have recently been reviewed [20]. In this study, we isolated three compounds from a marine-derived *Streptomyces* sp., identified their structures and investigated their MAO inhibitory activities, as well as the AChE, BChE, and BACE-1 inhibitory activities, for their possible multitarget inhibitions.

## Materials and Methods

### Structural Analysis

Optical rotations were acquired using a Kruss Optronic P-8000 polarimeter with a 5-cm cell. The ultraviolet (UV) spectra were measured with a V-730 UV-visible spectrophotometer (Jasco, USA) using a path length of 1 cm. The infrared spectra were recorded on a Varian Scimitar Series in CHCl_3_. The nuclear magnetic resonance (NMR) spectra were acquired at 300 MHz for ^1^H in CD_3_OD and DMSO-*d_6_* using a solvent signal as an internal reference (δ_H_ 3.31 and δ_H_ 2.50 for the respective solvents). Mass data were obtained on an Agilent Technologies 6120 quadrupole. Electrospray ionization mass spectroscopy (ESIMS) data were collected using an Agilent Technologies 6120 quadrupole mass spectrometer (Santa Clara, CA) coupled with an Agilent Technologies 1260 series HPLC with a reversed-phase column (Phenomenex Luna C-18(2) (100 Å, 50 mm × 4.6 mm, 5 μm) at a flow rate of 1.0 ml/min. The fractions were purified by a Waters 616 quaternary HPLC pump and a Waters 996 photodiode array detector using a Phenomenex Luna C-18(2) (250 mm × 10 mm, 5 μm) reversed HPLC column. HRMS analysis was conducted with a JEOL JMS-AX505WA mass spectrometer.

### Bacterial Culture and Isolation of Compounds

The marine-derived actinomycetes strain CNQ-031 was isolated from sediment sampled off the coast of California. It was identified as belonging to the *Streptomyces* sp. MAR4 clade on the basis of 16S rRNA gene sequence analysis. Strain CNQ-031 was cultured in 40 L of a 2.5-L ultra-yield flask containing 1 L of SYP SW medium (10 g/l of soluble starch, 2 g/l of yeast, 4 g/l of peptone, 10 g/l of CaCO_3_, 20 g/l of KBr, 8 g/l of Fe_2_(SO_4_)_3_ × 4(H_2_O) dissolved in 750 ml natural seawater and 250 ml of distilled water) at 25°C with shaking at 150 rpm for 7 days. The culture medium was extracted with EtOAc (40 L overall), and the EtOAc-soluble fraction was concentrated in vacuo to yield 5.84 g of crude extract. The entire crude extract was fractionated by C-18 open column chromatography with a step gradient from 20% to 100% MeOH in distilled water to obtain nine fractions. The third fraction was subjected to reversed-phase HPLC with 39% aqueous acetonitrile (Phenomenex Luna C-18(2) (100 Å, 250 mm × 100 mm, 2.0 ml/min, 5 μm) to obtain compound **1** (19.8 mg). The fourth fraction was chromatographed and eluted with 50% acetonitrile to isolate compound **3** (6.0 mg), and the fifth fraction was purified with 47% acetonitrile to provide compound **2** (11.4 mg).

### Chemicals and Enzyme Assays

Recombinant human MAO-A and human MAO-B, AChE from *Electrophorus electricus*, BChE from equine serum, the BACE-1 Activity Detection Kit, kynuramine, benzylamine, 5,5′-dithiobis(2-nitrobenzoic acid)(DTNB), acetylthiocholine iodide (ATCI), S-butyrylthiocholine iodide (BTCI), and reference reversible inhibitors (toloxatone, lazabemide, donepezil, and quercetin as inhibitors of MAO-A, MAO-B, AChE, BChE, and BACE-1, respectively) were purchased from Sigma-Aldrich (USA) [21]. The reference irreversible inhibitors (clorgyline and pargyline, inhibitors of MAO-A and MAO-B, respectively) were obtained from Bioassay Systems (USA) [22]. All other chemicals were of reagent grade.

MAO-A and MAO-B inhibitory activities were determined using a continuous spectrophotometric method as described previously [23, 24]. The K_m_ values of MAO-A for kynuramine and of MAO-B for benzylamine were 0.024 and 0.14 mM, respectively. The concentrations of kynuramine (0.06 mM) and benzylamine (0.3 mM) used were 1.5 and 1.8 times the respective K_m_ values. AChE activities were assayed as described by Ellman *et al*. [25], with slight modification [26, 27]. The inhibitory activities of AChE and BChE were measured after the enzyme was preincubated with inhibitors for 15 min and before adding DTNB and the substrate (ATCI and BTCI, respectively). Reactions were performed using approximately 0.2 U/ml of AChE and BChE in the presence of 0.5 mM DTNB and 0.5 mM substrate (ATCI and BChE) in 0.5-ml reaction mixtures; reactions were continuously monitored for 10 min at 412 nm. Reaction rates were expressed as changes in absorbance per minute [28]. BACE-1 assays were performed using the BACE-1 kit at 320 and 405 nm for excitation and emission wavelengths, respectively, and a fluorescence spectrometer (FS-2, Scinco, Korea) after reaction for 2 h at 37°C with 7-methoxycoumarin-4-acetyl-[Asn670,Leu671]-amyloid β/A4 protein fragment 667-676-(2,4-dinitrophenyl)Lys-Arg-Arg amide trifluoroacetate as a substrate [29].

### Analysis of Inhibitory Activities of the Isolated Compounds

Inhibition of MAOs, AChE, BChE, and BACE-1 by the three compounds was investigated at a concentration of 10 μM, and then IC_50_ values of the compounds and the reference inhibitors were determined. Kinetic parameters, inhibition types, and kinetic of inhibition (K_i_) values of potent MAO-A and MAO-B inhibitors (*i.e.*, compound **1** for MAO-A; compound **2** for MAO-A and MAO-B) were determined, as previously described [26]. The K_i_ values were measured at five different substrate concentrations for each MAO isoform (0.0075, 0.015, 0.03, 0.06, and 0.12 mM for MAO-A; 0.03, 0.06, 0.15, 0.3, and 0.6 mM for MAO-B) and in the absence or presence of each inhibitor at concentrations of approximately 0.5, 1.0, and 2.0 times their IC_50_ values [21]. Inhibitory patterns and K_i_ values were determined using Lineweaver-Burk plots and secondary plots of their slopes.

### Reversibility Tests of Compounds 1 and 2

The reversibilities of MAO inhibition by compounds **1** and **2** and by the reference compounds (toloxatone and clorgyline for MAO-A; lazabemide and pargyline for MAO-B) at twice the IC_50_ concentrations were investigated by dialysis as previously described [22]. After the compounds or reference inhibitors were preincubated with MAOs for 30 min, residual activities for undialyzed and dialyzed samples were measured. The relative values for undialyzed (A_U_) and dialyzed (A_D_) activities were then calculated, and the reversibilities were determined by comparing the A_U_ and A_D_ values of the inhibitors with those of the references.

## Results and Discussion

### Isolation and Identification of the Compounds

**Fig. 1:**
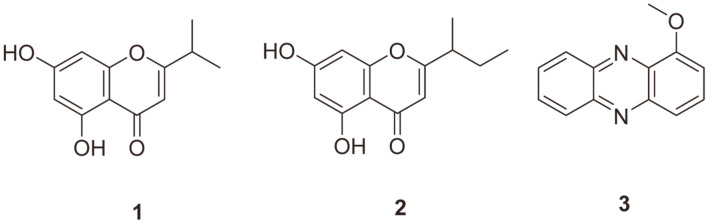
Structures of compounds 1, 2, and 3, which were isolated from *Streptomyces* sp. CNQ-031.

According to the procedure, three compounds were isolated, and their properties were as follows: compound **1** was a light-brown powder, LR-MS [M+H]^+^ *m/z* 221.1; compound **2** was a yellowish amorphous powder, LR-MS [M+H]^+^ *m/z* 235.2; and compound 3 was a yellow solid, LR-MS [M+H]^+^ *m/z* 211.1. The ^1^H NMR spectrum of compound **1** displayed a pair of meta-coupled aromatic protons [δ_H_ 6.33 (^1^H, d, *J* = 2.2 Hz, H-8) and 6.19 (^1^H, d, *J* = 2.2 Hz, H-6)], an olefinic proton [δ_H_ 6.05 (^1^H, s, H-3)], and an upfielded proton [δ_H_ 2.88 (^1^H, sext, *J* = 7.2 Hz, H-1′)]. The ^1^H NMR spectrum also displayed the two methyl singlets [δ_H_ 1.32 (3H, s, H-2′) and 1.30 (3H, s, H-3′)]. When the NMR data were compared with previous data, compound **1** was identified as 5,7-dihydroxy-2-isopropyl-4H-chromen-4-one [30]. The ^1^H NMR spectrum of compound **2** revealed a pair of meta-coupled aromatic protons [δ_H_ 6.33 (^1^H, d, *J* = 2.2 Hz, H-8) and 6.20 (^1^H, d, *J* = 2.2 Hz, H-6)], one olefinic proton [δ_H_ 6.05 (^1^H, s, H-3)], and three upfielded protons [δ_H_ 2.64 (^1^H, sext, *J* = 7.2 Hz, H-1′), 1.75 (^1^H, m, H-3′a), 1.64 (^1^H, m, H-3′b)]. The ^1^H NMR spectrum also displayed the two methyl singlets [δ_H_ 1.29 (3H, s, H-2′) and 0.94 (3H, t, *J* = 8.0 Hz, H-4′)]. The ^13^C NMR data of compound **2** showed 13 carbon signals, including two methyl carbons [δ_C_ 11.9 (C-4′), 18.2 (C-2′)], a methylene [δ_C_ 28.6 (C-3′)], an allylic methine [δ_C_ 41.7 (C-1′)], and six aromatic carbons [δ_C_ 94.8 (C-8), 100.1 (C-6), 107.6 (C-3), 159.9 (C-1a), 163.3 (C-5), and 166.1 (C-7)], together with an olefinic carbon [δ_C_ 105.4 (C-4a)], an oxygenated olefinic carbon [δ_C_ 175.8 (C-2)], and a conjugated carbonyl group [δ_C_ 184.1 (C-4)]. A comparison of the ^1^H and ^13^C NMR data to the previous data identified compound **2** as 5,7-dihydroxy-2-(1-methylpropyl)-4H-chromen-4-one [30]. The ^1^H NMR data of compound 3 showed seven aromatic protons in a 1,2,3-trisubstituted aromatic spin system [δ_H_ 7.53 (^1^H, dd, *J* = 7.47, 2.49 Hz, H-9), 7.42 (^1^H, dd, *J* = 7.47, 2.49 Hz, H-6), 7.16 (^1^H, m, H-7), 7.14 (^1^H, m, H-8)] and a 1,2,3,4-tetrasubstituted aromatic [δ_H_ 7.06 (^1^H, d, *J* = 8.22 Hz, H-4), 7.00 (^1^H, dd, *J* = 8.81, 1.49 Hz, H-3), 6.48 (^1^H, d, *J* = 7.47 Hz, H-2)], respectively. These aromatic protons revealed the presence of a phenazine moiety. The ^1^H NMR data also displayed a methoxy proton [δ_H_ 3.77 (3H, s, H-11)]. Compound **3** was identified as 1-methoxyphenazine according to a comparison of the ^1^H and ^13^C NMR data with previously reported data [31]. The structures of compounds **1**, **2**, and **3** are shown in Fig. 1.

### Inhibitory Activities of the Compounds

The three isolated compounds were assayed for inhibitory activity against MAOs, ChEs, and BACE-1. Compounds **1** and **2** showed residual activities of 25.4% and 37.8%, respectively, for MAO-A, and showed activities of 71.5% and 34.4%, respectively, for MAO-B at 10 μM (Table 1). Compound **3** showed little inhibitory activity against MAO-A and MAO-B. The IC_50_ of compound **1** for MAO-A was 2.70 μM, and the selectivity index (SI) was 10.0 versus MAO-B (IC_50_ of compound 1 against MAO-B = 27.0 μM); the IC_50_ of compound 2 for MAO-B was 3.42 μM, and the SI was low (2.02) versus MAO-A (IC_50_ of compound 2 against MAO-A = 6.92 μM) (Table 1). The 1-methylpropyl group in compound **2** had increased MAO-B inhibitory activity compared with the isopropyl group in compound **1**. Compound **3**, which had a phenazine instead of a chromenone scaffold, was not effective at inhibiting MAO-A or MAO-B. All three compounds showed little inhibitory activity against AChE, BChE, and BACE-1; the highest IC_50_ value for compound **1** (31.7 μM) was against AChE (Table 1). These results indicated that compound **1** was a potent and selective inhibitor of MAO-A and that compound **2** was a potent and low-selective inhibitor of MAO-B. No tested compounds showed multitargeting inhibitory activities.

Compound **1** in this study was a potent and selective MAO-A inhibitor (IC_50_ = 2.70 μM; SI = 10.0), whereas a piloquinone isolated from a marine source in another study was a potent and selective MAO-B inhibitor (IC_50_ = 1.21 μM; SI = 6.47) [17]. Compared with other natural inhibitors from microbial sources, compound 1 displayed higher inhibitory activity against MAO-A than did an anithiactin (IC_50_ = 13.0 μM) [18], HMC (IC_50_ = 13.97 μM)[19], and UroA or Ur°C (IC_50_ = 5.88 or 29.6 μM, respectively) [32], but it displayed lower inhibitory activity compared with an aplysinopsin (IC_50_ = 0.0056 μM) [16], UroB (IC_50_ = 0.88 μM) [32], and an alternariol monomethyl ether (IC_50_ = 1.71 μM) [33]. Compound **2** was a low-selective MAO-B inhibitor (IC_50_ = 3.42 μM; SI = 2.02), and it displayed inhibitory activity against MAO-B similar to that of HMC (IC_50_ = 3.23 μM) [19] but lower inhibitory activity than that of a piloquinone (IC_50_ = 1.21 μM) [17]. All three tested compounds had low inhibitory activity against AChE, BChE, and BACE-1.

### Reversibilities of Compounds 1 and 2

**Fig. 2:**
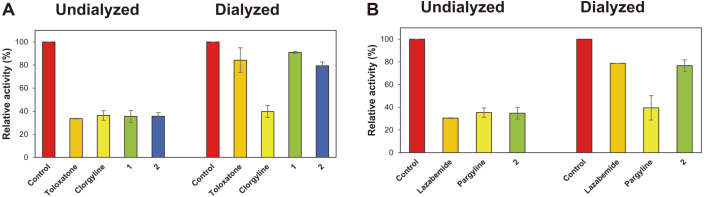
Recovery of monoamine oxidase (MAO)-A inhibition by compounds 1 and 2 (A) and of MAO-B inhibition by compound 2 (B) after dialysis. To study MAO-A inhibition, toloxatone (2.16 μM) and clorgyline (0.014 μM) were used as the reference reversible and irreversible inhibitors, respectively, for compounds **1** (5.40 μM) and **2** (13.8 μM). To study MAO-B inhibition, lazabemide (2.20 μM) and pargyline (0.28 μM) were used as the reference reversible and irreversible MAO-B inhibitors, respectively, for compound **2** (6.84 μM). Results are the averages of experiments performed in duplicate.

In reversibility experiments by dialysis, inhibition of MAO-A by compounds **1** and **2** was substantially recovered, from 35.5% (A_U_) to 90.9% (A_D_) and from 35.6% to 79.4%, respectively (Fig. 2A). These values were similar to those observed for the reversible inhibitor toloxatone (33.6% to 84.2%). Little recovery was observed for the irreversible inhibitor clorgyline (36.3% to 39.7%) (Fig. 2A). Inhibition of MAO-B by compound **2** was recovered from 34.8% (A_U_) to 76.7% (A_D_), and these recovery values were similar to those observed for the reversible inhibitor lazabemide (30.5% to 78.7%), whereas little recovery was observed for the irreversible inhibitor pargyline (35.4% to 39.5%) (Fig. 2B). These results indicated that compound **1** was a reversible inhibitor of MAO-A and that compound **2** was a reversible inhibitor of MAO-A and MAO-B.

### Kinetics of Compounds 1 and 2

**Fig. 3:**
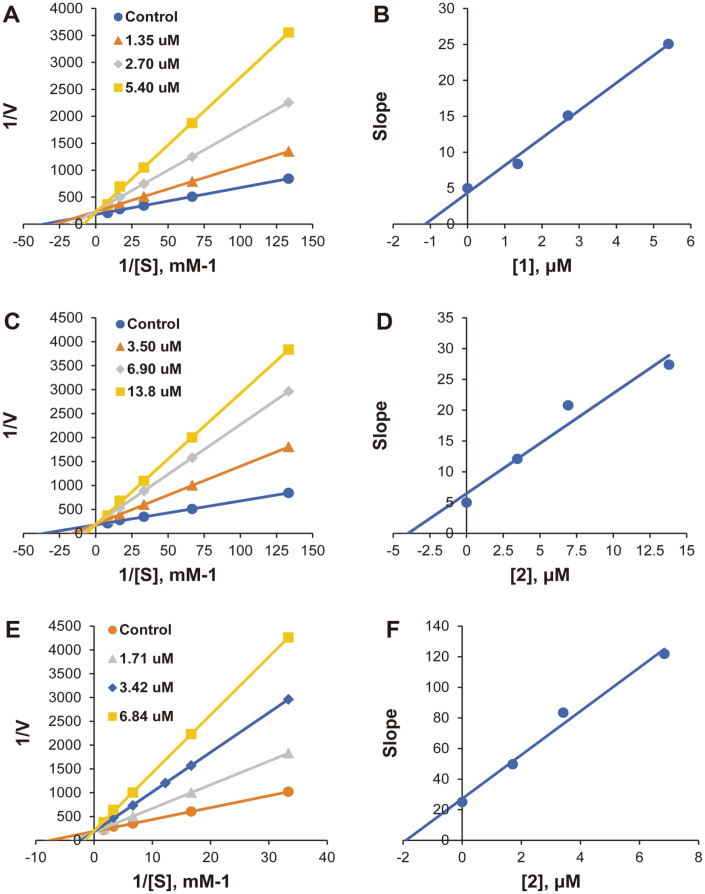
Lineweaver-Burk plots of monoamine oxidase (MAO)-A inhibition by compound 1 (A) and of MAOA and MAO-B inhibition by compound 2 (C and E for respective MOA isoforms) as well as their respective secondary plots, as slopes of Lineweaver-Burk plots vs. inhibitor concentrations (B, D, and F). Substrate MAOA and MAO-B concentrations ranged from 0.0075 to 0.12 and from 0.03 to 0.6 mM, respectively. Experiments were carried out at three inhibitor concentrations: ~0.5, 1.0, and 2.0 times the 50% inhibitory concentration (IC_50_). The initial velocity was expressed as an increase in absorbance per min.

Modes of MAO-A inhibition by compound **1** and modes of MAO-A and MAO-B inhibition by compound **2** were investigated using Lineweaver-Burk plots. Plots of MAO-A inhibition by compound **1** were linear and intersected the *y*-axis (Fig. 3A), indicating that compound 1 is a competitive inhibitor of MAO-A. Secondary plots of the slopes of the Lineweaver-Burk plots against the inhibitor concentrations showed a K_i_ value of 0.94 ± 0.28 μM for compound **1** (Fig. 3B). Similarly, compound 2 was a competitive inhibitor of MAO-A and MAO-B (Figs. 3C and 3E); its K_i_ values were 3.57 ± 0.60 and 1.89 ± 0.014 μM, respectively (Figs. 3D and 3F).

In this study, three compounds—two chromenones (compounds **1** and **2**) and one phenazine (compound **3**)—were isolated from marine-derived *Streptomyces* sp. CNQ-031. Compound **1** potently and selectively inhibited MAO-A, and compound **2** inhibited MAO-B and next MAO-A with low selectivity. Compound **3** showed little inhibitory activity against MAO-A and MAO-B. All three compounds weakly inhibited AChE, BChE, and BACE-1. Compound **1** was a reversible competitive inhibitor of MAO-A, and compound **2** was a reversible competitive inhibitor of MAO-A and MAO-B; thus, both compound **1** and compound **2** are effective MAO inhibitors from a microbial source.

## Figures and Tables

**Table 1:** Inhibition of MAO-A, MAO-B, AChE, BChE, and BACE-1 by the compounds isolated from *Streptomyces* sp. CNQ-031^a^.

Compounds	Residual activity at 10 µM (%)					IC_50_ (µM)					**SI** ^b^
	
	MAO-A	MAO-B	AChE	BChE	BACE-1	MAO-A	MAO-B	AChE	BChE	BACE-1	
**1**	25.4 ± 2.24	71.5 ± 5.45	70.5 ± 6.31	80.7 ± 3.46	85.8 ± 0.28	2.70 ± 0.034	27.0 ± 2.78	31.7 ± 2.39	-	-	10.0
**2**	37.8 ± 0.82	34.4 ± 7.95	88.3 ± 2.54	96.4 ± 4.27	98.3 ± 0.27	6.92 ± 0.067	3.42 ± 1.47	-	-	-	2.02
**3**	93.6 ± 4.11	96.9 ± 2.65	83.0 ± 8.24	97.0 ± 2.72	79.9 ± 0.73	-	-	-	-	-	
Toloxatone						1.08 ± 0.025	-	-	-	-	
Lazabemide						-	0.11 ± 0.016	-	-	-	
Clorgyline						0.0070 ± 0.0007	-	-	-	-	
Pargyline						-	0.14 ± 0.0059	-	-	-	
Donepezil						-	-	0.0095 ± 0.0019	0.18 ± 0.0038	-	
Quercetin						-	-	-	-	13.4 ± 0.035	

^a^Results are the means ± SD of duplicate experiments.

^b^SI values were given as the ratio of high IC_50_/low IC_50_ for MAO inhibition.
